# Oral Microbiome in Health and Disease: Maintaining a Healthy, Balanced Ecosystem and Reversing Dysbiosis

**DOI:** 10.3390/microorganisms11061453

**Published:** 2023-05-31

**Authors:** Tom Maier

**Affiliations:** Division of Biomaterial and Biomedical Sciences, Department Oral Rehabilitation and Biosciences, School of Dentistry, Oregon Health and Sciences University, Portland, OR 97201, USA; maiert@ohsu.edu

The oral microbiome is a complex and dynamic assemblage of microorganisms that normally exist within the mouth, contributing to host health via a number of mechanisms, including exclusion of harmful microbes and immune optimization [1,2,3].

Oral dysbiosis refers to an imbalance or disruption in this community, which can arise from various factors such as disease, poor oral hygiene, medication use, and diet. Dysbiosis of the oral microbiome can contribute to the development of numerous oral and systemic diseases, including dental caries [4,5], periodontitis [5,6], oral and other cancers [7,8,9], cardiovascular disease [10,11], and diabetes [12]. It is associated with an increase in pathogenic bacteria and a corresponding decrease in beneficial bacteria. This understanding has led to extensive research focused on interventions to restore microbial health and balance in the oral microbiome, including prebiotics, probiotics, and antimicrobial agents, as potential therapeutic approaches for treating and preventing oral dysbiosis and its associated diseases.

This Special Issue, The Oral Microbiome in Systemic Health and Disease: Therapeutics for Reversing Microbial Dysbiosis and Applications in Precision Medicine, aims to define the role of the oral microbiome in systemic health and disease, as well as to describe therapies that can potentially reverse or rebalance the oral microbiome towards health. The issue includes nine articles, comprising four research and five review articles, addressing these issues.

Alqaderi et al. [13] investigated the diversity of the salivary microbiome in Kuwaiti adolescents with different body mass indexes (BMI). They collected saliva samples from adolescents of normal weight, overweight, and obesity and used next-generation sequencing to analyze the microbiota. The results showed that the salivary microbiome diversity differed significantly between the three groups, with the overweight and obese adolescents having less diversity compared to those with normal weight. This study adds to a growing body of data suggesting that salivary microbiome diversity could be a potential biomarker for assessing the risk of obesity in adolescents.

Two studies, one by Fukuda et al. [14], the other by Mocanu et al. [15], examined ways to change dysbiotic microbiomes, one in vitro and the other in vivo.

The article by Fukuda et al. [14] used deep ultraviolet light-emitting diode (DUV-LED) therapy in vitro to target Fusobacterium nucleatum, a common oral bacterium associated with both oral and systemic diseases, including colon cancer and Alzheimer’s dementia. The results showed that DUV-LED therapy at a wavelength of 285 nm significantly reduced the viability of *F. nucleatum*. The study suggests that DUV-LED therapy could be a potential treatment for oral diseases caused by *F. nucleatum*, such as periodontitis and halitosis, and by extension perhaps some systemic diseases as well. 

The article by Mocanu et al. [15] describes a randomized clinical trial that investigated the microbiological profiles of patients with dental prosthetic treatment and periodontitis before and after photoactivation therapy. These researchers collected samples from the patients’ gums and prosthetic restorations and analyzed the microbiota using culture-dependent and culture-independent methods. The results showed that photoactivation therapy significantly reduced the number of microorganisms and altered the composition of the microbiota in both the gums and prosthetic restorations. The study suggests that photoactivation therapy could be an effective adjunctive treatment for patients with dental prosthetic treatment and periodontitis. 

The last research article by Rozas et al. [16] discussed the oral factors that can influence the composition of oral microbiota in individuals with Parkinson’s Disease. These factors include saliva production, oral hygiene, and the use of medications such as anticholinergics. It was suggested that a dysbiotic imbalance of oral microbiota may contribute to the development/progression of Parkinson’s Disease and its associated symptoms and emphasized the importance of proper oral care and regular dental checkups to help maintain a healthy oral biofilm in individuals with Parkinson’s Disease.

The five review articles examine the potential impact of the microbiome on systemic health, covering topics such as infective endocarditis, mental health, cancer, gastro-intestinal diseases, and chronic diseases such as cardiovascular diseases and diabetes.

In their review, Vyhnalova et al. [17] discuss the potential role of the oral microbiota in the development and progression of oral squamous cell carcinoma (OSCC). The authors review various studies that have identified changes in the oral microbiota associated with OSCC and suggest that these alterations may contribute to the etiopathogenesis of OSCC. They discuss the potential mechanisms by which the oral microbiota could promote OSCC, including the induction of chronic inflammation and the production of carcinogenic metabolites. The article underscores the importance of further research to better understand the role of the oral microbiota in OSCC and to identify potential oral microbiome therapeutic targets for the prevention and treatment of this disease.

Maitre et al. [18] present an intriguing review of the potential for prebiotics and probiotics to modulate the oral microbiota and their potential as therapeutic adjuncts in mental disorders. The authors analyze various studies that suggest a link between the gut microbiota and mental health and propose that the oral microbiota may also play a role in this relationship. They discuss the potential mechanisms by which prebiotics and probiotics could influence the oral microbiota, such as by promoting the growth of beneficial bacteria and reducing the abundance of pathogenic bacteria. The article suggests that prebiotics and probiotics could be promising adjunctive therapies for mental disorders, particularly those associated with dysbiosis of the oral and gut microbiota. 

The link between the oral microbiome and infective endocarditis (IE) has been well established, and Del Giudice et al. [19] review how this knowledge can be used to help prevent this serious disease. IE is a potentially life-threatening infection of the heart valves or lining, which can be caused by bacteria from various sources, including the oral cavity. The authors analyze various studies that show a link between oral bacteria and IE and discuss the potential mechanisms by which oral bacteria can cause IE, such as through the formation of biofilms and the production of virulence factors. The article emphasizes the importance of oral health in maintaining a healthy oral microbiome to reduce the risk of IE, especially for individuals at high risk of IE. 

There is a growing awareness of the link between gut microbiome dysbiosis and gastro-intestinal diseases. Contaldo et al. [20] extend this idea and explore the relationship between the oral microbiota and salivary levels of oral pathogens and gastrointestinal (GI) diseases. The authors review existing literature on the subject and conduct an exploratory study to investigate the oral microbiota and salivary levels of oral pathogens in patients with various GI diseases, such as inflammatory bowel disease (IBD) and celiac disease. The study finds that patients with IBD had a different oral microbiota composition and higher levels of oral pathogens in their saliva compared to healthy individuals. The study also finds a positive correlation between the severity of GI symptoms and the abundance of oral pathogens in saliva. The article suggests that oral pathogens may play a role in the etiology and progression of GI diseases and emphasizes the importance of maintaining a healthy oral microbiome in the prevention and management of these conditions. The article concludes by suggesting that further research is needed to better understand the relationship between the oral microbiota and GI diseases and to develop more effective prevention and treatment strategies.

The final review by Khor, Snow et al. [21] explores the potential links between the oral and gut microbiomes, and their impact on systemic health and disease. The authors conduct a comprehensive review of the existing literature on the subject and discuss the potential mechanisms by which the oral and gut microbiomes interact, including the transfer of oral bacteria to the gut and modulation of immune function. The article suggests that dysbiosis of both the oral and/or gut microbiome can have systemic effects on health, including the development of chronic diseases such as cardiovascular disease and diabetes. The review emphasizes the importance of maintaining a balanced and diverse oral and gut microbiome, as dysbiosis of either can have systemic effects on health, including the development of chronic diseases like cardiovascular disease and diabetes. The authors suggest that probiotics and prebiotics may have potential as therapeutic interventions for restoring microbial balance and improving systemic health. Finally, the review concludes by highlighting the need for further research to better understand the complex interconnections between the oral and gut microbiomes and their impact on local and systemic health and disease.
